# SGK1, a Critical Regulator of Immune Modulation and Fibrosis and a Potential Therapeutic Target in Chronic Graft-Versus-Host Disease

**DOI:** 10.3389/fimmu.2022.822303

**Published:** 2022-02-10

**Authors:** Run-qing Lu, Yin-yin Zhang, Hai-qiu Zhao, Rong-qun Guo, Zhong-xing Jiang, Rong Guo

**Affiliations:** Department of Hematology, The First Affiliated Hospital of Zhengzhou University, Zhengzhou, China

**Keywords:** serum/glucocorticoid regulated kinase 1 (SGK1), graft-versus-host disease (GVHD), Th2 cell, Th17 cell, fibrosis, transplant, autoimmune

## Abstract

Patients with severe chronic graft-versus-host disease (cGVHD) always experience debilitating tissue injury and have poorer quality of life and shorter survival time. The early stage of cGVHD is characterized by inflammation, which eventually leads to extensive tissue fibrosis in various organs, such as skin and lung, eventually inducing scleroderma-like changes and bronchiolitis obliterans syndrome. Here we review the functions of serum/glucocorticoid regulated kinase 1 (SGK1), a hub molecule in multiple signal transduction pathways and cell phosphorylation cascades, which has important roles in cell proliferation and ion channel regulation, and its relevance in cGVHD. SGK1 phosphorylates the ubiquitin ligase, NEDD4, and induces Th cells to differentiate into Th17 and Th2 phenotypes, hinders Treg development, and promotes inflammatory fibrosis. Phosphorylation of NEDD4 by SGK1 also leads to up-regulation of the transcription factor SMAD2/3, thereby amplifying the fibrosis-promoting effect of TGF-β. SGK1 also up-regulates the inflammatory transcription factor, nuclear factor-κB (NF-κB), which in turn stimulates the expression of multiple inflammatory mediators, including connective tissue growth factor. Overexpression of SGK1 has been observed in various fibrotic diseases, including pulmonary fibrosis, diabetic renal fibrosis, liver cirrhosis, hypertensive cardiac fibrosis, peritoneal fibrosis, and Crohn’s disease. In addition, SGK1 inhibitors can attenuate, or even reverse, the effect of fibrosis, and may be used to treat inflammatory conditions and/or fibrotic diseases, such as cGVHD, in the future.

## Introduction

With the increasing clinical application of haploidentical hematopoietic stem cell transplantation, the incidence of chronic graft-versus-host disease (cGVHD) is increasing annually. Transplant recipients with cGVHD have a reduced quality of life and an increased risk of long-term morbidity and mortality compared with those without cGVHD (1). Patients with mild or moderate cGVHD may have longer survival due to a lower relapse rate, but patients with severe cGVHD have poorer quality of life and shorter survival time. The manifestations of cGVHD are heterogeneous and involved in most tissues. The typical performances include skin lichenoid plaques and sclerosis, sicca symptoms in the eyes, fibrosis in joints, skin and lung. It is generally recognized that the pathophysiological processes of cGVHD include three phases: (a) early inflammation due to tissue injury, (b) thymic injury and T cell and B cell dysregulation, and (c) fibrosis (2). The above stages can exist independently or overlap with others. Not all stages must occur nor do they have to happen sequentially (3). The early stage of cGVHD is characterized by inflammation, which leads to extensive tissue fibrosis and even severe disability (2). More than 20% of patients with cGVHD will experience sclerosis, which is characterized by thickening of the skin, or fasciitis caused by collagen deposition and fibrosis (4, 5). Fibrosis in various tissues, such as skin and lung, eventually leads to scleroderma-like changes and bronchiolitis obliterans syndrome (BOS). As the main infiltrating inflammatory cells, helper T (Th) cells can differentiate into Th1, Th2, Th17, or regulatory T (Treg) cells. Chronic GVHD is mainly characterized by Th17 other than Th1-skewed responses, immune dysregulation and/or fibrosis, which is immunologically different from acute GVHD (6). However, the signaling pathways and molecular mechanisms that mediate the imbalance in Th cell differentiation remain unclear.

## Overview of Serum/Glucocorticoid Regulated Protein Kinase 1

SGK1 is a member of the protein kinase subfamily, and a serine/threonine protein kinase with high homology to second messengers, such as protein kinase B (PKB/Akt) (7). SGK1 also has two specific Ser/Thr regulatory sites: Thr256 in its catalytic domain and the Ser422 in its C-terminus. SGK1 is distributed in the lung, kidney, heart, liver, and other tissues. Although its expression levels are very low in most cells, it is sensitive to external stimuli. When cells are stimulated by glucocorticoid or serum, *SGK1* gene expression increases rapidly by 5-10-fold within 30 minutes; hence, it was named as serum/glucocorticoid regulated protein kinase. SGK1 has a short mRNA half-life (around 20 min) and its expression and activity are regulated by a variety of stimuli through transcriptional, translational and post-translational mechanisms (8). Its transcription is stimulated by dehydration and a modest increase of extracellular salt concentration, excessive glucose concentration, high-salt diet, etc (9–12). SGK1 transcription is also stimulated by some mediators like glucocorticoids, mineralocorticoids, transforming growth factor b (TGFβ), interleukin-6, fibroblast and platelet-derived growth factor (8, 13). SGK1 translation is triggered by phosphoinositide 3 kinase (PI3K) and dependent on actin polymerization and regulated by several mechanisms including TGFβ-dependent transcription factors SMAD3 and SMAD4 (14). SGK1 is ubiquitinated by Nedd4-2 and Rictor/Cullin-1, which trigger SGK1 degradation (15). As a hub of multiple signal transduction pathways and cell phosphorylation events, SGK1 plays important roles in cell proliferation, ion channel regulation, signal transduction, and other physiological processes, and is recognized as having significant functions in inflammation.

## Mechanisms Underlying SGK1 Activity in Inflammatory and/or Fibrotic Diseases

### SGK1 Regulation of the Th1/Th2 Inflammatory Axis

**Figure 1:**
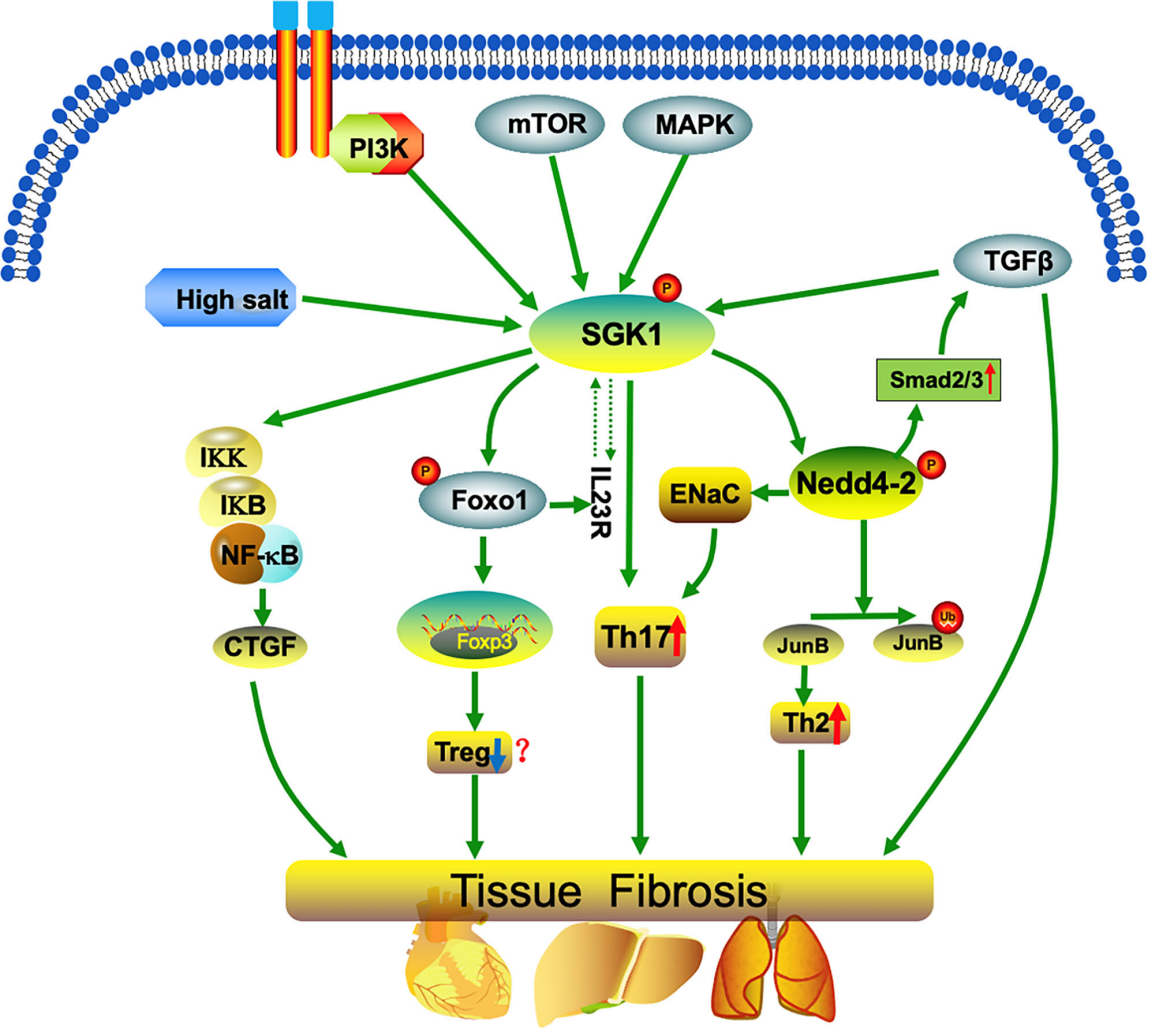
SGK1 mediates imbalanced differentiation of Th17 and Th2 cells. SGK1 regulation occurs both at the level of transcription (for example, by a high-salt diet, p38MAPK, or TGF-β) and at the post-translational level. SGK1 phosphorylates the ubiquitin ligase, NEDD4, and induces Th cell differentiation into Th17 and Th2 phenotypes, hinders Treg development (promotes Treg differentiation in a few studies), and promotes up-regulation of the transcription factor, SMAD2/3, thereby amplifying the pro-fibrotic effects of TGF-β. Further, SGK1 up-regulates the inflammatory transcription factor, nuclear factor-κB (NF-κB), which in turn stimulates the expression of multiple inflammatory mediators, including connective tissue growth factor (CTGF). Together, the processes described above promote the occurrence of inflammation and fibrosis.

Th2 type T cells secret IL-4 to activate the proliferation of B cells to promote B cell isotype switching, causing a series of pathological changes, including autoantibody secretion and collagen deposition, which are part of the mechanisms underlying the development of cGVHD (3). SGK1 is activated by the continuous phosphorylation of two specific serine/threonine (Ser/Thr) sites, which involves phosphorylation by PI3K-dependent protein kinase at Thr256 and by mTORC2 at Ser422 (16). mTOR can form two different protein complexes: mTORC1 and mTORC2. Genetic deletion of the mTORC2 adaptor protein, Rictor, leads to dysfunction of Th2 cells, including inability to produce IL-4 (17). mTORC2 promotes Th cell differentiation into the Th2 phenotype through the SGK1-ubiquitin ligase NEDD4-2-JunB pathway (18) (**Figure 1**). Luo et al. (19) also demonstrated that SGK1 kinase activation promotes Th cell differentiation to the Th2 phenotype. In a mouse model of allergic asthma, SGK1 deficiency significantly reduces Th2 cell differentiation, while bronchoalveolar lavage fluid from *Sgk1* gene knockout mice has lower concentrations of IL-4 and IgE, and mice were, therefore, resistant to Th2 cell-mediated allergic asthma (20).

### SGK1 Regulation of the Th17/Treg Inflammatory Axis

Th17 cells and an activated Th17-prone, CD146-expressing, CD4+ T-cell subset participate in the development of cGVHD in the BO mouse model (21). By secreting IL-17, Th17 cells promote the proliferation and migration of neutrophils, the activation of endothelial cells, and the proliferation of fibroblasts, which is crucial in the BOS response following transplantation (22, 23). During BOS development after transplantation, down-regulation of Th17 cells can reduce damage of bronchioles. Granulocyte colony-stimulating factor (G-CSF)-mobilized donor grafts cause murine cGVHD with prominent scleroderma and high levels of Th17 cells, which recruit macrophages and produce higher level of profibrotic TGF-β, which are essential for lung and skin fibrosis (24, 25).

SGK1 is regulated at the transcription level (for example, by high-salt diet and p38MAPK) and at the post-translational level (for example, by PI3K). Further, SGK1 inhibits sodium channel degradation by phosphorylating the ubiquitin ligase, NEDD4-2, thereby promoting cell absorption of Na^+^ (26). SGK1 activates epithelial cell sodium channels in response to stimulation by high salt concentration, and promotes IL-23R expression to mediate Th17 cell differentiation. SGK1 is also regulated by PI3K phosphorylation, which promotes Th17 cell differentiation (**Figure 1**), hinders Treg cell formation, and accelerates autoimmune disease development (27). Further, the harmful effects of high salt concentrations on Th cell differentiation can be reversed by SGK1 inhibitors (10). Hence, SGK1 is a key kinase that induces Th17 cell differentiation, and p38/MAPK, NFAT5, or SGK1 gene silencing inhibit Th17 cell differentiation induced by high salt (28).

Recent studies have shown that SGK1 regulates the balance between Th17 and Treg cells, and that deficiency of SGK1 can correct autoimmune diseases caused by Th17/Treg ratio imbalance (9, 27). SGK1 can phosphorylate and inactivate FOXO1, preventing it from binding to FOXP3, thereby restricting FOXP3 expression and hindering Treg cell expansion (27). Treg cells cultured in high salt concentrations have a pro-inflammatory phenotype, characterized by increased IFN-γ secretion, that leads to loss of Treg cell function (29). Feeding a high-salt diet to mice following allogeneic heart transplantation accelerated heart transplantation rejection, and the harmful effects of a high-salt diet on Treg cells in transplanted mouse were offset by absence of SGK1 (30). On the other hand, it was also reported that the salt-SGK1 signaling axis endows Treg cells a Th17-like RORγt+ Foxp3+ phenotype *in vitro* and *in vivo*. These functionally specialized Treg cells are adaptive to high salt conditions and maintain their suppressive functions. The exact function of RORγt+ Foxp3+ Treg cells in inflammation need to be clarified (9). These results suggest that the effects of SGK1 on Treg need further investigation.

### Fibrogenic Effects of SGK1

The hallmark of fibrosis in cGVHD is aberrant tissue repair promoted by activated macrophages that produce TGF-β and platelet-derived growth factor α (PDGF-α), which leads to fibroblast activation. Extracellular matrix collagen and biglycan are produced by the activated fibroblasts, which cross-link collagen and increase tissue stiffness (6). Tissue fibrosis and macrophage activation have been reported in patients with chronic GVHD (31, 32). Pathogenic Th17 cells are observed in patients with lichenoid chronic GVHD and contribute to the development of chronic disease (33). Increased Th17/Treg ratio plays an important role in liver fibrosis formation in cGVHD (34). By secreting of IL-17, Th17 cells can mobilize, activate, and recruit neutrophils, promote neutrophil proliferation and migration, and influence the activation of endothelial cells, as well as activation and proliferation of fibroblasts (35). The characteristic of Th2 cells is the production of signature cytokines including IL-4, IL-5 and IL-13. Th2 cells together with eosinophils, basophils, macrophages, and type 2 innate lymphoid cells (ILC2) participate in the pathological process of Th2 immunity-induced fibrosis (36). SGK1 promotes Th cell differentiation to Th2 and Th17 phenotypes, and limits Treg proliferation. In addition, SGK1 expression is up-regulated after induction by the powerful fibrosis-promoting factor, TGFβ. The increase in cell volume caused by TGF-β1 contributes to the formation of fibrosis, and SGK1 promotes sodium ion influx to increase cell volume and accelerate fibrosis formation. Waldegger et al. cultured human intestinal mucosa, liver cancer cell lines, and the U937 cell line *in vitro* and found that SGK1 can be upregulated by TGF-β1 transcription (37). Further, Waerntges et al. demonstrated that TGF-β1 can up-regulate SGK1 expression in human lung fibroblasts, and that this can be partially reversed by p38 kinase antagonists (38). High concentrations of TGFβ in the target tissues of patients with cGVHD contribute to scleroderma and BOS development, and reducing TGFβ production, may improve organ function and reverse cGVHD fibrosis (32, 39, 40).

TGF-β-stimulated fibrosis is partly mediated by upregulation of the transcription factor, SMAD2/3, which is degraded by the ubiquitin ligase, NEDD4L (8). SGK1 phosphorylates NEDD4L, which prevents its interaction with SMAD2/3, thereby amplifying the pro-fibrotic effects of TGF-β (8). Further, SGK1 activates nuclear factor-κB (NFκB), mediating inflammation and fibrosis (13). Overexpression of SGK1 phosphorylates and activates IKK, which in turn phosphorylates IκB, the inhibitor of NFκB, triggers IκB degradation, releases NFκB inhibition, and improves its activity and nuclear translocation (13). Further, it stimulates the expression of a variety of inflammatory mediators, including connective tissue growth factor (CTGF) (13, 41). SGK1-dependent expression of CTGF assists in mineralocorticoid-stimulated cardiac fibrosis and skin aging (13, 42). Overexpression of SGK1 has been observed in various fibrotic tissues, including pulmonary fibrosis, diabetic renal fibrosis, liver cirrhosis, and hypertensive cardiac fibrosis, and SGK1 inhibitors can significantly reduce the degree of fibrosis in various tissues, suggesting that SGK1 has an important role in the development of inflammation and fibrosis (43–48).

### SGK1 Inhibitors

SGK1 has been found to participate in multiple pathological conditions and it may serve as a potential therapeutic target in various diseases. Research efforts have been made to develop SGK1 inhibitors that block or inhibit its activity. The pyrrolo-pyridine compound GSK650394 is a potent SGK1 inhibitor, developed by GlaxoSmithKline that has a higher selectivity of more than thirty folds for SGK1 over AKT and other related kinases (49). But GSK650394 has been reported to have comparable potency for some other kinases and it is not totally selective of SGK1 (50). GSK650394 has been shown to delay cancer progression by inhibiting cell growth and inducing apoptosis in prostate cancer cells (49, 51, 52). The epithelial-mesenchymal transition of renal tubular epithelial cells is inhibited by GSK650394 in the diabetic nephropathy mice (53). The brain ischemic area decreases in a mouse model and the reduction of cortisol-induced neurogenesis is avoided in hippocampal progenitor cells with the administration of GSK650394, suggesting its therapeutic potential in stress and depression (54, 55).

SGK1 inhibitor EMD638683 is developed by Merck with an IC50 of 3 μM *in vitro* and demonstrated efficacy *in vivo (* 56). EMD638683 inhibit cardiac fibrosis and remodeling through attenuating cardiac inflammation in an angiotensin II infusion-induced hypertension mouse model (48). Macrophage infiltration and pulmonary arterial smooth muscle cell proliferation are inhibited by EMD638683 treatment in the lungs of rats with pulmonary arterial hypertension (57). EMD638683 reduces vascular calcification and stiffness in cholecalciferol-overloaded chronic kidney disease mouse model (58). EMD638683 reverses glucose absorption in a mouse model, suggesting that SGK1 may serve as a therapeutic target in metabolic disorders (59).

SGK1 inhibitors SI113 is one of pyrazolopyrimidine-based derivatives with an IC50 value of 600nM (60). It has been shown to induce cytotoxic autophagy and inhibit cell growth in glioblastoma cells and endometrial cancer cells, suggesting its therapeutic potential in cancer treatment (61–63).

In summary, SGK1 inhibitors have been developed and studied in various pathological conditions. These compounds have shown significant inhibitory effects, suggesting their potential value of clinical application in multiple diseases, including carcinomas, metabolic disorders, brain ischemia and depression, etc. SGK1 inhibitors also exhibit potent inhibitory effects in fibrotic diseases, and their therapeutic potential in cGVHD need to be studied in the future since fibrosis is one of the major phases in the pathogenesis of cGVHD.

## Prospects

A high-salt diet can increase SGK1 expression, and dietary factors are becoming an area of considerable interest in the field of inflammation and/or fibrosis research. The high-salt/SGK1/TGF-β pathway is expected to become a new direction in investigations into the pathogenesis of cGVHD. At present, the specific mechanism by which SGK1 leads to organ fibrosis is not well understood, but may be related to the following mechanisms: 1) SGK1 can promote Th cell differentiation into Th17 and Th2 cells; 2) SGK1 is a transcription target of TGF-β, leading to fibrosis, and is closely related to organ fibrosis; and 3) SGKl may mediate the process of fibrosis by downstream regulation of CTGF.

Whether the frequent administration of glucocorticoids in the treatment of aGVHD and/or cGVHD might exacerbate the development of fibrosis though SGK1-mediated mechanisms remains little studied. Until now, the investigation of the role of SGK1 in transplantation immunity remains limited, especially in the overlap syndromes, which should be further explored in the future.

In summary, SGK1 has important roles in fibrosis formation and development, and has potential to become a new therapeutic molecular target. Research into SGK1 is a promising new avenue in investigation of the pathogenesis, prevention, and treatment of cGVHD.

## Author Contributions

R-QL, Y-YZ, and H-QZ are responsible for the collection, collation, and writing of the original manuscript. R-QG, Z-XJ, and RG are responsible for the concept development, revision, and review of the manuscript. All authors contributed to the article and approved the submitted version.

## Funding

This work was supported by Natural Science Foundation of Henan Province (182300410301), Medical Science and Technology Research Project Of Henan Province (SBGJ202102147, SBGJ202003036, 2018020118).

## Conflict of Interest

The authors declare that the research was conducted in the absence of any commercial or financial relationships that could be construed as a potential conflict of interest.

## Publisher’s Note

All claims expressed in this article are solely those of the authors and do not necessarily represent those of their affiliated organizations, or those of the publisher, the editors and the reviewers. Any product that may be evaluated in this article, or claim that may be made by its manufacturer, is not guaranteed or endorsed by the publisher.
